# PBK attenuates paclitaxel‐induced autophagic cell death by suppressing p53 in H460 non‐small‐cell lung cancer cells

**DOI:** 10.1002/2211-5463.12855

**Published:** 2020-04-14

**Authors:** Jung‐Hwan Park, Sang‐Ah Park, Young‐Ju Lee, Hwan‐Woo Park, Sang‐Muk Oh

**Affiliations:** ^1^ Department of Biochemistry College of Medicine Konyang University Daejeon Korea; ^2^ Department of Cell biology College of Medicine Konyang University Daejeon Korea; ^3^ Priority Research Center Myunggok Medical Research Institute College of Medicine Konyang University Daejeon Korea

**Keywords:** Mdm2, non‐small‐cell lung cancer cells, p53, PBK, ubiquitination

## Abstract

PDZ‐binding kinase (PBK) has previously been shown to mediate chemoresistance of cancer cells to anticancer drugs. However, it remains unclear how PBK regulates paclitaxel‐induced cancer cell death. Here, we demonstrate that PBK hinders paclitaxel‐mediated autophagic cell death in H460 non‐small‐cell lung cancer cells. PBK knockdown increased apoptosis, autophagy, p53 level, and LC3 puncta upon paclitaxel treatment. Moreover, p53 expression facilitated an increase in the LC3‐II/LC3‐I ratio in response to paclitaxel, and PBK knockdown augmented paclitaxel‐mediated p53 transcriptional activity. Meanwhile, paclitaxel induced PBK‐mediated p53 nuclear export and its subsequent ubiquitination in control cells, but not in PBK knockdown cells. We conclude that PBK hampers paclitaxel‐induced autophagic cell death by suppressing p53, suggesting a potential role of PBK in p53‐mediated H460 cell death.

## Graphical abstract

When PDZ‐binding kinase (PBK) is present (B), PBK interacts with p53 in the nucleus and then translocates to the cytoplasm upon paclitaxel treatment, leading to Mdm2‐mediated p53 ubiquitination and subsequent degradation, which induces a decrease in p53‐mediated apoptosis or autophagy in H460 non‐small‐cell lung cancer cells. In contrast, p53‐mediated apoptosis and autophagy are markedly promoted in the absence of PBK (A).

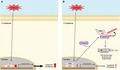

**Abbreviations**
AMPK: AMP‐activated protein kinase
CDK: cyclin‐dependent kinase
FIP200: FAK family‐interacting protein of 200 kDa
LC3: microtubule‐associated protein 1A/1B‐light chain 3
MAPK: mitogen‐activated protein kinase
Mdm2: mouse double minute 2 homolog
mTOR: mammalian target of rapamycin
PARP: poly (ADP‐ribose) polymerase
PBK: PDZ‐binding kinase
siRNA: small interfering RNA
TRAIL: TNF‐related apoptosis‐inducing ligand

Paclitaxel, a natural product extracted from the bark of the Pacific Yew (*Taxus brevifolia*), has been commonly used as chemotherapeutic agent for a variety of cancer including breast, ovarian, and non‐small‐cell lung cancer, bladder, head and neck, prostate, nasopharyngeal carcinoma, and esophageal cancers [1, 2, 3]. It has been shown that paclitaxel binds to beta‐tubulin, promotes microtubule stability, and inhibits microtubule disaggregation leading to cell death by cell cycle arrest in M phase [4]. Meanwhile, cyclin‐dependent kinase‐1 (Cdk1)/cyclin B1 was shown to be activated by continuous M phase in paclitaxel treatment [5, 6]. In addition, paclitaxel is known to result in apoptosis either via Raf‐1 kinase activation or tumor suppressor p53 depending on the dose concentration [1, 7, 8, 9]. In addition, it has been suggested that paclitaxel induces autophagy [10, 11]. However, correlation between paclitaxel‐induced apoptosis and autophagy remains to be unclear. Autophagy plays a key role in maintenance of cellular homeostasis and cell viability under various stresses [12]. Generally, autophagy is known to eliminate protein aggregates and damaged mitochondria. However, prolonged autophagy can induce cell death via inordinate self‐digestion or activation of apoptosis. Several studies have suggested that survival autophagy occurs in response to starvation and some chemotherapeutic drugs involving tamoxifen or cisplatin [13, 14, 15]. Meanwhile, autophagic cell death has been shown to occur in response to hypoxia, oxidative stress, radiation, lipopolysaccharide (LPS), and some chemotherapeutic drugs involving rapamycin and endostatin [16, 17, 18, 19, 20]. Several reports demonstrate that nuclear p53 potentiates autophagy [13, 21, 22]. On the other hand, cytoplasmic p53 is known to suppress autophagy. That is, p53 has distinct role depending on its subcellular localization, cytoplasm, or nucleus [23, 24, 25]. However, the autophagy mechanism regulated by p53 is not yet clear.

PDZ‐binding kinase (PBK) is highly expressed in many cancer cells and is known as MAPKK‐like protein kinase [26, 27, 28, 29, 30]. Functionally, PBK has been shown to function as an upstream effector for p38 mitogen‐activated protein kinase (MAPK), MKP1, and peroxiredoxin 1 (Prx1) [26, 31, 32]. Moreover, both JNK1 in UVB‐mediated cell transformation and ERK2 during tumorigenesis were upregulated by upstream kinase PBK [33, 34]. In addition, expression of PTEN known as suppressor in PI3K‐AKT signaling pathway is blocked by PBK. The cyclin B1/Cdk1 acts an upstream molecule for PBK during mitosis, thereby promoting cytokinesis [27, 35]. It has been suggested that PBK binds to DNA‐binding domain on p53 and represses p53 transcriptional activity, leading to a decrease in DNA damage‐induced apoptosis through reduction of p21 expression [36]. We previously reported that PBK confers TRAIL or doxorubicin resistance to HeLa cervical cancer cells by regulating IκBα phosphorylation [37, 38].

We first reveal that PBK is a novel target molecule in paclitaxel‐mediated signaling to induce autophagic cell death. We also demonstrate that PBK is phosphorylated on Thr9 residue and p53 expression is increased in response to paclitaxel, and that p53 plays a key role in paclitaxel‐induced autophagy. Moreover, we indicate that in the presence of PBK, p53 nuclear export and subsequent Mdm2‐mediated ubiquitination occur during paclitaxel treatment. These findings provide evidence that PBK plays a key role in paclitaxel induction of p53‐mediated autophagic or apoptotic cell death of non‐small‐cell lung cancer cells.

## Materials and methods

### Cell culture and reagents

Human embryonic kidney 293 (HEK 293) cells and human non‐small‐cell lung carcinoma (H460) cells were purchased from American Type Culture Collection (ATCC, Manassas, VA, USA). HEK293 or H460 cell line was maintained in DMEM or RPMI 1640 supplemented with 10% FBS, 2 mm l‐glutamine, and 1% penicillin/streptomycin, respectively. Paclitaxel, bafilomycin A1, nutlin‐3, anti‐LC3 antibody, and anti‐β‐actin antibody were purchased from Sigma (St. Louis, MO, USA). Z‐VAD‐FMK was purchased from Selleckchem (Houston, TX, USA). HI‐PBK 032 was purchased from Tocris Bioscience (Bristol, UK). Anti‐PBK, anti‐p53, and anti‐Mdm2 antibodies were purchased from Abcam (Cambridge, MA, USA). Anti‐p‐PBK (Thr‐9) and anti‐cleaved PARP antibodies were purchased from Cell Signaling Technology, Inc. (Beverly, MA, USA). Anti‐GFP and anti‐HA antibodies were purchased from Santa Cruz Biotechnology, Inc. (Santa Cruz, CA, USA). Alexa Fluor^®^ 488 goat anti‐rabbit IgG, Alexa Fluor^®^ 594 goat anti‐rabbit IgG, Alexa Fluor^®^ 488 goat anti‐mouse IgG, and Alexa Fluor^®^ 594 goat anti‐mouse IgG antibodies were purchased from Thermo Fisher Scientific (Waltham, MA, USA). Transfection reagents, Effectene, or Lipofectamine 3000 were purchased from Qiagen, Inc. (Valencia, CA, USA) or Invitrogen (Grand Island, NY, USA), respectively.

### Plasmids and transfection

PBK siRNA or control siRNA construct was as described previously [37]. pGL2‐p21 promoter‐Luc was a gift from M. Walsh (Addgene plasmid #33021) [39]. pGL2‐356bp was a gift from W. EI‐Deiry (Addgene plasmid #16292) [40]. pcDNA3 MDM2 WT was a gift from M.‐C. Huung (Addgene plasmid #16233) [41]. pRK5‐HA‐Ubiquitin‐WT was a gift from T. Dawson (Addgene plasmid #17608) [42]. P53‐GFP was a gift from G. Wahl (Addgene plasmid #11770). GFP‐LC3 was a gift from J. Debnath (Addgene plasmid #22405). 293 or H460 cells growing on 100‐mm dishes were transfected with each of plasmid using Effectene or Lipofectamine 3000 according to the manufacturer's instructions. Twenty‐four after transfection, cells were lysed, and cell lysate was subjected to immunoblotting or immunoprecipitation.

### Immunoblotting and immunoprecipitation

For immunoblot analysis, 30 μg of each total cell lysate was resolved on SDS/PAGE, probed with each antibody, and images were achieved with SuperSignal West Pico Chemiluminescent Substrate (Pierce Biotechnology, Inc., Rockford, IL, USA) and X‐ray film. For immunoprecipitation, 500 μg of total cell lysate was incubated with indicated antibody and protein agarose beads at 4 °C for 2 h using rocker. The beads were washed, mixed with sample buffer, boiled for 5 min, and subjected to 10% SDS/PAGE and then immunoblotting.

### Luciferase assay

Stable control siRNA cells or PBK siRNA cells growing on six‐well plates were transfected with each 1 µg of p53 or p21 promoter linked to luciferase reporter plasmids plus 0.5 µg of the *pRL‐SV40* gene using Lipofectamine 3000. Twenty‐four after transfection, cells were treated with paclitaxel for 24 h, harvested, and then lysed. Firefly and Renilla luciferase activities were measured and normalized using cell lysate.

### Confocal microscopy

Control cells or PBK knockdown cells growing on six‐well plates were transfected with GFP‐p53, GFP‐LC3, or V5‐PBK constructs using Lipofectamine 3000. Twenty‐four after transfection, cells were treated with paclitaxel for indicated time. Paclitaxel‐treated cells were fixed in 4% paraformaldehyde for 15 min at room temperature, and then washed with ice‐cold PBS. Next, cells were permeabilized with 0.25% Triton X‐100 and then blocked with 1% BSA for 30 min at room temperature. Fixed cells were incubated with primary antibodies during overnight at 4 °C, washed, and then stained with 1 : 200 diluted Alexa Fluor 488 or 594 antibodies. Nuclei were counterstained with 4′,6‐diamidino‐2‐phenylindole dihydrochloride (DAPI). Images were acquired using confocal microscopy.

### Flow cytometry analysis

Briefly, control cells or PBK knockdown cells growing on 60‐mm dishes at a density of 2 × 10^6^ cells were treated with each inhibitor, Z‐VAD‐FMK or nutlin‐3, for 2 h, and then, paclitaxel was added. After incubation, apoptosis was analyzed with flow cytometry (FACSCalibur, BD Biosciences, San Jose, CA, USA) using the Annexin V‐FITC and propidium iodide according to the manufacturer's instruction (Thermo, Waltham, MA, USA).

### Cell viability assay

Cell viability was determined via 2‐(2‐methoxy‐4‐nitrophenyl)‐3‐(4‐nitrophenyl)‐5‐(2,4‐disulfophenyl)‐2H‐tetrazolium (WST‐8) assay. Control or PBK knockdown of NCI‐H460 cells was seeded in 96‐well plates at 5 × 10^3^ cells/well. After 24 h, the cells were treated with inhibitors, such as HI‐PBK 032, bafilomycin A1, or Z‐VAD‐FMK, incubated for 2 h, after which 10 μL of WST‐8 was added to each well and incubated for 4 h at 37 °C, and then, the absorbance was determined at 450 nm.

### Colony‐forming assay

A transformation assay of H460 cells was carried out. Briefly, H460 cells were seeded in 6‐well plates at a density of 1 × 10^4^ cells. After 24 h, cells were treated with inhibitors, such as Z‐VAD‐FMK, bafilomycin A1, or nutlin‐3 during 2 h, and then, paclitaxel was added for 24 h. Foci were stained with 0.5% crystal violet, and then, the number of colonies was counted under a microscopy.

### Statistical analysis

Results are indicated as the mean ± standard deviation (SD) for at least three independent experiments in duplicates. Statistical analysis was done by two‐tailed Student's *t*‐test or one‐way ANOVA. *P* values less than 0.05 were considered as significant.

## Results

### Depletion or inhibition of PBK increases paclitaxel‐induced H460 cell death

**Fig. 1:**
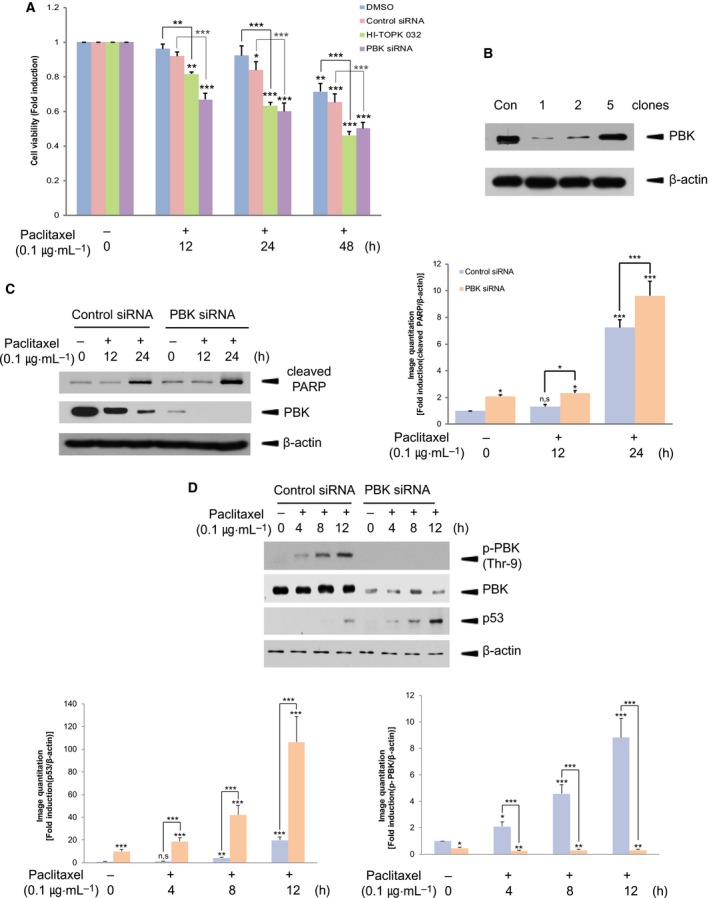
Inhibition of PBK expression or activity promotes paclitaxel‐induced H460 cell death. (A) H460 cells were treated with DMSO and 0.1 μg·mL^−1^ of paclitaxel alone or together with HI‐TOPK 032 (3 μm) for indicated time, or were transfected with control siRNA or PBK siRNA, and then incubated with paclitaxel. Cell viability was measured by WST‐1 cell proliferation assay. (B) Control siRNA cells (Con) or stable PBK siRNA cells (1, 2, 5 clones) were established using G418. Immunoblotting was done to verify PBK expression. (C) Control siRNA cells or stable PBK siRNA cells were treated with DMSO or paclitaxel (0.1 μg·mL^−1^) for 12 or 24 h. Immunoblot analysis was performed using PBK or cleaved PARP antibody. (D) Control siRNA cells or stable PBK siRNA cells were incubated with DMSO or paclitaxel (0.1 μg·mL^−1^) for indicated time. Cell lysates were subjected to immunoblotting using indicated antibody. Endogenous β‐actin level was used for loading control. Representatives of three independent experiments are shown. *P < 0.05; **P < 0.01; and ***P < 0.001 compared to controls. Statistical analysis was done by two‐tailed Student's *t*‐test.

We have suggested that PBK plays a key role in TRAIL or doxorubicin resistance of human HeLa cervical cancer cells [37, 38]. In this report, we first asked whether expression or activity of PBK affected one of the anticancer drugs, paclitaxel‐induced death of non‐small‐cell lung cancer cell line H460. H460 cells were treated with paclitaxel plus vehicle, DMSO, or PBK inhibitor, HI‐TOPK 032 for indicated time, respectively. Also, cells were transfected with control siRNA or PBK siRNA, and treated with paclitaxel 48 h after transfection. As expected, cell viability was decreased in response to paclitaxel in time‐dependent manner (Fig. 1A). Interestingly, PBK inhibitor or PBK siRNA promoted paclitaxel‐induced cell death. This finding indicated that PBK might play a pivotal role in chemoresistance against paclitaxel in non‐small‐cell lung cancer cells. We next generated stable PBK knockdown H460 cells using PBK siRNA. The desired clone (clone #1) was selected and used for further experiments (Fig. 1B). Paclitaxel treatment of stable PBK knockdown cells resulted in much more increase in cleaved poly (ADP‐ribose) polymerase (PARP), compared with control knockdown cells (Fig. 1C), suggesting involvement of PBK in paclitaxel‐mediated apoptotic pathway. Meanwhile, paclitaxel induced phosphorylation on threonine 9 residue of PBK in control knockdown cell but not PBK knockdown cells time‐dependently (Fig. 1D). CDK1/cyclin B1 in M phase of cell cycle is known to act as an upstream effector that phosphorylates threonine 9 residue of PBK [43]. Also, paclitaxel has been suggested to activate CDK1/cyclin B1 [44, 45]. Together, paclitaxel‐induced phosphorylation on threonine 9 residue of PBK might be due to activated CDK1/cyclin B1. It is reported that PBK binds to p53 and suppresses p53 expression [36]. We found that endogenous p53 level was greatly increased by paclitaxel treatment in PBK knockdown cells, compared with control cells (Fig. 1D), suggesting PBK's regulatory role in p53 expression in response to paclitaxel.

### PBK knockdown augments paclitaxel‐induced autophagic cell death

**Fig. 2:**
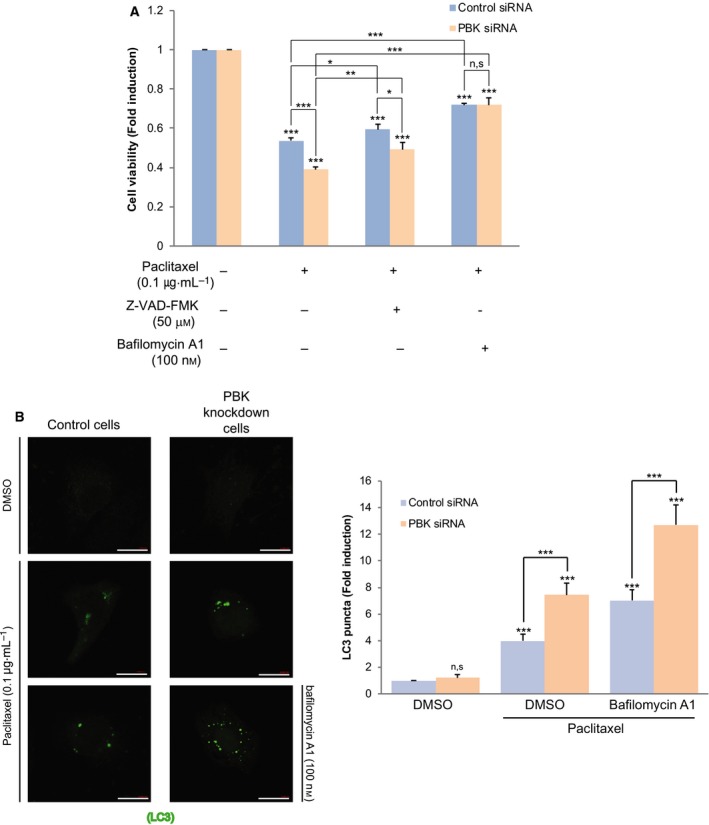
Depletion of PBK increases autophagic cell death in response to paclitaxel. (A) Control siRNA cells or PBK siRNA cells H460 cells were treated with DMSO, and paclitaxel (0.1 μg·mL^−1^) alone or in combination with Z‐VAD‐FMK (50 μm) or bafilomycin A1 (100 nm) for 24 h, and then, cell viability was examined. (B) Control cells or PBK knockdown cells were transfected with GFP‐LC3 construct. Twenty‐four hours after transfection, cells were incubated with paclitaxel (0.1 μg·mL^−1^) alone or paclitaxel plus bafilomycin A1 (100 nm) for 24 h. GFP‐LC puncta (green) was observed by confocal microscopy. Scale bar, 20 μm. Representative images of 63× magnification from three independent experiments are indicated. *P < 0.05; **P < 0.01; and ***P < 0.001 compared to controls. N.S, nonsignificant. Statistical analysis was performed by one‐way ANOVA.

We next examined whether paclitaxel‐mediated cell death depends on caspase‐mediated apoptosis. Control cells or PBK knockdown cells were treated with paclitaxel alone or paclitaxel plus pan‐caspase inhibitor, Z‐VAD‐FMK. Unexpectedly, the cell viability was a little restored by caspase inhibitor (Fig. 2A). We questioned whether autophagy is responsible for paclitaxel‐mediated cell death. The cells were treated with paclitaxel alone or paclitaxel plus autophagosome inhibitor, bafilomycin A1. Interestingly, autophagosome inhibitor considerably improved the cell viability (Fig. 2A). These results showed that paclitaxel‐induced cell death relies on autophagy and apoptosis. We next investigated the effect of paclitaxel on expression of autophagic marker LC3. GFP‐LC3 construct was transfected into control cells or PBK knockdown cells, treated with combination of paclitaxel or bafilomycin A1. As shown in Fig. 2B, LC3 puncta was increased in PBK knockdown cells, compared with control cells. Bafilomycin A1 enhanced the LC3 puncta. These findings demonstrate that PBK ablation promotes paclitaxel‐mediated autophagic cell death.

### PBK alleviates paclitaxel induction of p53‐mediated autophagy and negatively regulates p53 function

**Fig. 3:**
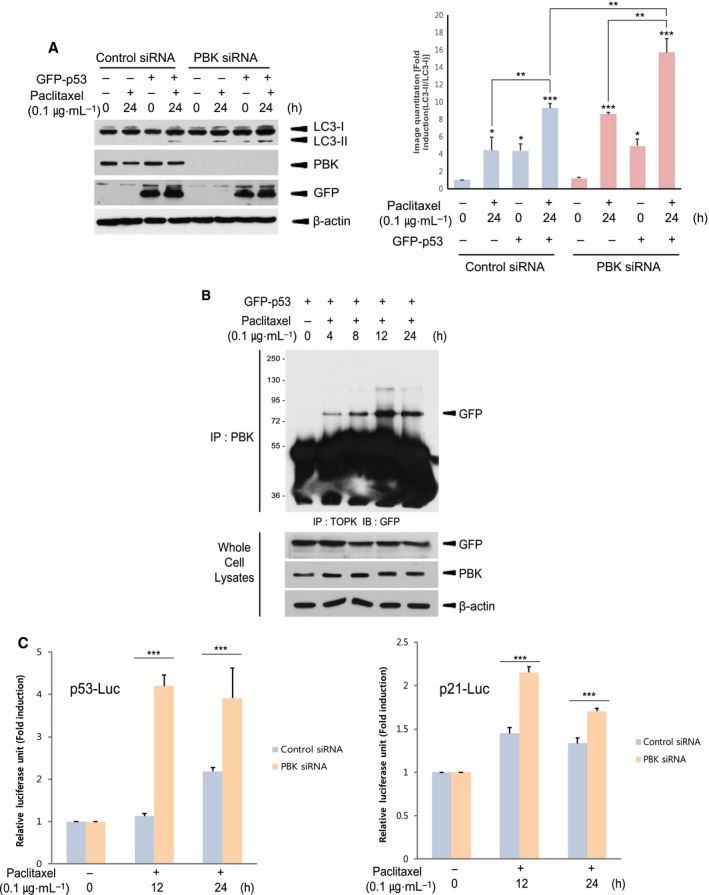
PDZ‐binding kinase diminishes p53‐mediated both autophagy and transcriptional activity induced by paclitaxel. (A) Control siRNA cells or PBK siRNA cells were transfected with GFP‐p53. Twenty‐four hours after transfection, cells were treated with DMSO or paclitaxel (0.1 μg·mL^−1^) for 24 h. Cell lysates were subjected to SDS/PAGE and immunoblotting using indicated antibody. Representatives of three independent experiments and graph for quantitation are shown. (B) H460 cells were transfected with GFP‐p53 construct. Twenty‐four hours after transfection, cells were incubated with DMSO or paclitaxel (0.1 μg·mL^−1^) for indicated time. Immunoprecipitation using PBK antibody and protein A/G plus‐agarose bead was done, and then, immunoblotting was performed using GFP antibody. GFP‐p53 expression or endogenous PBK level was confirmed using cell lysate. (C) Control siRNA cells or PBK siRNA cells were transfected with p53 (*p53‐LUC*) or p21 promoter (*p21‐LUC*)‐driven luciferase reporter constructs plus *pRL‐SV40* gene. Twenty‐four hours after transfection, cells were incubated with DMSO or paclitaxel (0.1 μg·mL^−1^) for 24 h. Firefly luciferase activity was normalized against *Renilla* luciferase activity. *P < 0.05; **P < 0.01; and ***P < 0.001 compared to controls. Results are indicated as the mean ± standard deviation for at least three independent experiments in duplicates. Statistical analysis was performed by two‐tailed Student's *t*‐test.

p53 is well known to be tumor suppressor and regulate apoptosis in cancer cells treated with anticancer drugs such as paclitaxel, cisplatin, and doxorubicin. In addition, it has been suggested that p53 regulates autophagy in oncogenic stressed cancer cells [13, 21, 22]. We next investigated the effect of p53 on paclitaxel‐induced autophagy. Control cells or PBK siRNA cells were transfected with empty vector or GFP‐p53, and then incubated with DMSO or paclitaxel for 24 h. The results showed that exogenous expression of p53 significantly elevated amount of LC3‐II itself and paclitaxel‐induced LC3‐II, and that depletion of PBK enhanced paclitaxel induction of p53‐mediated autophagy (Fig. 3A). On the other hand, PBK was shown to bind to p53 and suppress p53 expression [36]. Based on this report, we explored whether PBK could associate with p53 and regulate p53 transcriptional activity in response to paclitaxel. To examine the interaction between PBK and p53, H460 cells were transfected with GFP‐p53 construct and incubated with paclitaxel or DMSO for indicated time. The results indicated that paclitaxel considerably increased the association of PBK with p53 (Fig. 3B). Also, control cells or PBK siRNA cells were transfected with p53 promoter or p53 target, p21 promoter‐driven luciferase reporter construct, and treated with paclitaxel or DMSO for 12 or 24 h. As expected, p53 or p21 transcriptional activity induced by paclitaxel was substantially increased in PBK knockdown cells compared with control cells (Fig. 3C). Together, these findings demonstrate that PBK can interact with p53 and negatively regulate p53 expression or activity, leading to a decrease in p53‐mediated autophagy in response to paclitaxel.

### PBK mediates paclitaxel‐induced p53 ubiquitination

**Fig. 4:**
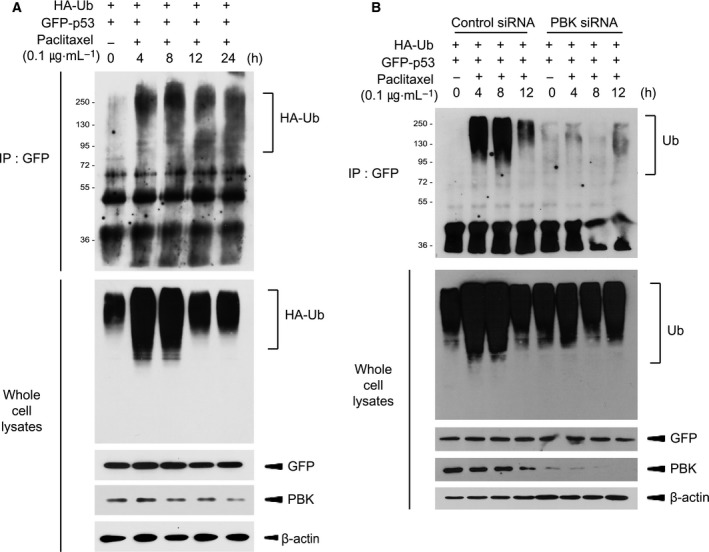
PBK is required for paclitaxel‐induced p53 ubiquitination. (A) 293 cells were transfected with GFP‐p53 together with HA‐Ub construct and were incubated with DMSO or paclitaxel (0.1 μg·mL^−1^) at 24 h post‐transfection for indicated time. Cell lysate was subjected to immunoprecipitation using GFP antibody and protein A/G plus‐agarose bead, and then, immunoblotting was done using HA antibody. Also, cell lysate was probed with indicated antibody. (B) Control siRNA cells or PBK siRNA cells were transfected with GFP‐p53 plus HA‐ubiquitin (Ub) construct and were treated with DMSO or paclitaxel (0.1 μg·mL^−1^) 24 h after transfection for indicated time. Immunoprecipitation using GFP antibody and subsequent immunoblotting using HA antibody was performed. Cell lysate was subjected to immunoblotting using indicated antibody. Shown are representatives of three independent experiments.

**Fig. 5:**
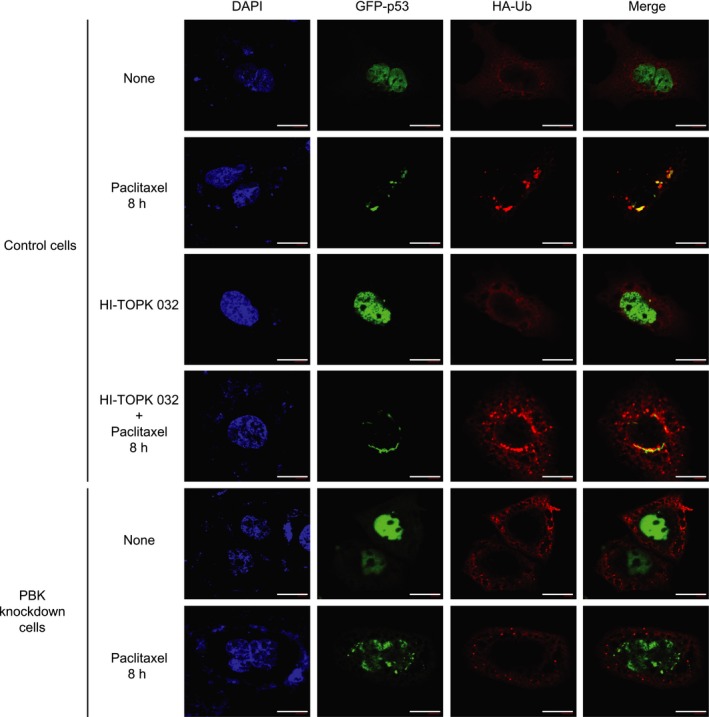
PDZ‐binding kinase knockdown blocks paclitaxel‐mediated translocation of nuclear p53 to cytoplasm, leading to failure to its subsequent ubiquitination. Control cells or PBK knockdown cells were cotransfected with GFP‐p53 and HA‐Ub construct. Twenty‐four hours after transfection, cells were treated with DMSO, paclitaxel (0.1 μg·mL^−1^) alone, and HI‐TOPK 032 (3 μm) alone or in combination of paclitaxel with HI‐TOPK 032 for 8 h. Cells were immunostained with HA (*HA‐Ub*; red) antibody. The nucleus is stained with DAPI (blue). Merged images (merge) are indicated. Scale bar, 20 μm. Shown images are representatives of 63× magnification from three independent experiments. Results are shown as the mean ± standard deviation for at least three independent experiments in duplicates.

As shown in Fig. 3, PBK interacted with p53 and alleviated p53 promoter activity. These findings might be due to p53 degradation that is mediated by PBK. Therefore, we next investigated whether p53 ubiquitination occurs in response to paclitaxel and depends on PBK. 293 cells were transfected with HA‐ubiquitin construct together with GFP‐p53 construct, and then treated with paclitaxel or DMSO. Paclitaxel treatment resulted in a dramatic increase in p53 ubiquitination (Fig. 4A). Also, HA‐ubiquitin construct plus GFP‐p53 construct were expressed in control cells or PBK siRNA cells, and then incubated with paclitaxel or DMSO for indicated time. Interestingly, paclitaxel‐induced p53 ubiquitination was greatly abolished in PBK knockdown cells (Fig. 4B). It is well known that cytoplasmic p53 located in cytoplasm suppresses autophagy, while nuclear p53 that remains in nucleus increases the expression of autophagy‐related proteins leading to autophagy [23, 25]. Based on these previous reports, we explored subcellular localization of p53 in control cells or PBK knockdown cells during paclitaxel treatment. Confocal microscopy data demonstrated that paclitaxel treatment of control cells for 8 h induced translocation of GFP‐p53 from nucleus to cytoplasm where p53 was ubiquitinated (Fig. 5). PBK inhibitor, HI‐TOPK 032, did not affect the p53 location. However, GFP‐p53 continuously stayed at nucleus in PBK knockdown cells despite paclitaxel treatment. These results indicate that the reason why p53‐mediated autophagy is reduced in cells expressing PBK upon paclitaxel treatment might be due to cytoplasmic localization, ubiquitination, and subsequent degradation of p53 dependent on PBK expression.

### Inhibition of Mdm2 elevates paclitaxel‐mediated autophagy or apoptosis

**Fig. 6:**
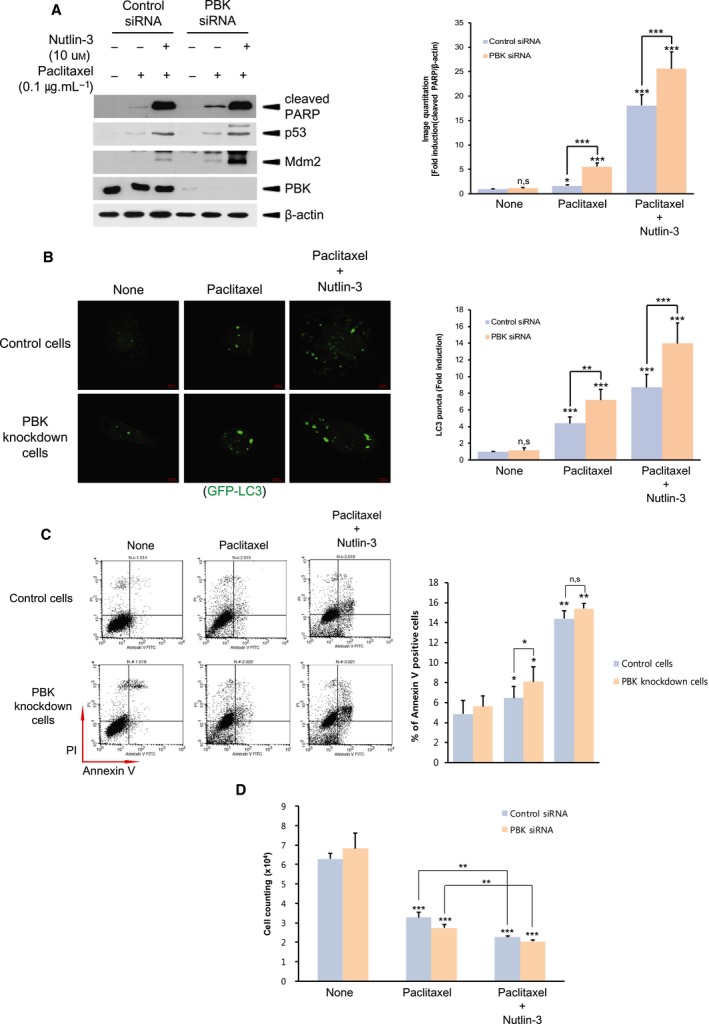
Inhibition of interaction of Mdm2 with p53 promotes both autophagy and apoptosis during paclitaxel treatment. (A) Control siRNA cells or PBK siRNA cells were treated with DMSO, and paclitaxel (0.1 μg·mL^−1^) alone or in combination with nutlin‐3 (10 μm) for 24 h. Cell lysate was subjected immunoblotting using indicated antibody. (B) Control cells or PBK knockdown cells were transfected with GFP‐LC and 24 h after transfection, cells were incubated DMSO, and paclitaxel (0.1 μg·mL^−1^) alone or in combination with nutlin‐3 (10 μm) for 24 h. GFP‐LC puncta was observed by confocal microscopy. Representatives of 63× magnification from three independent experiments are indicated. Scale bar, 20 μm. (C) Control cells or PBK knockdown cells were treated with DMSO, and paclitaxel (0.1 μg·mL^−1^) alone or in combination with nutlin‐3 (10 μm) for 24 h. Apoptosis was analyzed by Annexin V‐FITC and propidium iodide staining, and subsequent flow cytometry. Representatives of three independent experiments and graph for quantitation of Annexin V‐positive cells are shown. (D) Control siRNA cells or PBK siRNA cells were incubated with DMSO, and paclitaxel (0.1 μg·mL^−1^) alone or in combination with nutlin‐3 (10 μm) for 24 h. Cell counting was done by colony‐forming assay. *P < 0.05; **P < 0.01; and ***P < 0.001 compared to controls. Results are indicated as the mean ± SD for at least three independent experiments in duplicates. Statistical analysis was done by two‐tailed Student's *t*‐test.

Previous report suggests that p53 in the nucleus is transferred to the cytoplasm, subjected to Mdm2‐mediated ubiquitination and subsequent 26S proteasome‐mediated degradation [46]. We next investigated the effect of inhibition of Mdm2 on paclitaxel‐mediated autophagy or apoptosis. Nutlin‐3, an inhibitor of interaction between p53 and Mdm2, was employed to prevent Mdm2‐mediated p53 ubiquitination and degradation. As shown in Fig. 6A, treatment of nutlin‐3 markedly increased paclitaxel‐induced p53 or cleaved PARP level. Also, Mdm2 level was elevated by nutlin‐3, which results from the fact that Mdm2 is one of the transcription factor p53‐dependent genes. Moreover, nutlin‐3 augmented paclitaxel‐induced GFP‐LC3 puncta, which might be due to an increased p53 level (Fig. 6B). Agreeing with Fig. 6A, flow cytometry analysis or colony‐forming assay indicated that paclitaxel‐mediated apoptosis or cell growth was substantially promoted or alleviated upon nutlin‐3 treatment, respectively (Fig. 6C,D). These results show that Mdm2 plays a regulatory role in paclitaxel induction of p53‐mediated autophagy or apoptosis‐derived H460 cell death.

## Discussion

Our previous studies have suggested that PBK confers chemoresistance against anticancer drugs such as doxorubicin or TRAIL on HeLa cervical cancer cells [37, 38]. However, information about signaling pathways or stimuli involving anticancer drugs that regulate PBK activity or expression to affect cancer cells still lacks. In this study, we demonstrate that PBK diminishes p53‐mediated non‐small‐cell lung cancer cell death upon treatment of paclitaxel, a mitotic inhibitor used in cancer chemotherapy. It has been proposed that paclitaxel inhibits the disassembly of microtubule to block spindle formation, leading to G2/M phase cell cycle arrest [47]. In continuous M phase, CDK1/cyclin B1 was shown to be activated during paclitaxel treatment [47, 48, 49, 50]. Also, CDK1/cyclin B1 in M phase of cell cycle phosphorylates threonine 9 residue of PBK [43]. Interestingly, we found that phosphorylation on threonine 9 of PBK was induced during paclitaxel treatment, which might be due to paclitaxel‐mediated CDK1/cyclin B1 activation.

Paclitaxel was shown to induce not only apoptosis but also autophagy [51, 52] and to cause p53 expression that serves as a key tumor suppressor protein in response to various cellular death stimuli [53, 54, 55]. It has been suggested that autophagy is a highly conserved catabolic process with critical functions in maintenance of cellular homeostasis under normal growth conditions or stress [56]. The autophagy‐induced cell death is known as type II program cell death [57, 58]. Also, p53 is shown to be deeply implicated in both apoptosis and autophagy. Nuclear p53 induces autophagy and transcription of several apoptotic genes related to apoptosis. Meanwhile, cytoplasmic p53 suppresses autophagy by FIP200 or AMPK/mTOR pathway [59]. We indicated that PBK depletion increased paclitaxel‐induced H460 cell death, but inhibitor of caspase or autophagy decreased the cell death. In addition, endogenous p53 level was time‐dependently increased in PBK knockdown cells upon paclitaxel treatment, implying correlation between PBK and p53 on paclitaxel exposure. Moreover, expression of exogenous p53 elevated GFP‐LC3 puncta. Together, our results demonstrate that endogenous PBK negatively regulates paclitaxel‐induced cell death including both apoptosis and autophagy, and p53 plays a pivotal role in the cell death in response to paclitaxel.

Previous studies have suggested that PBK suppresses p53 expression or activity through binding to DNA‐binding domain of p53 [36, 60, 61]. However, how p53 expression is regulated by PBK is still not understood. We found that PBK bound to p53 and paclitaxel‐mediated p53 or p21 transcriptional activity was promoted in PBK knockdown cell compared with control cells. More importantly, paclitaxel induced p53 translocation from nucleus to cytoplasm and ubiquitination in PBK‐expressing cells but not PBK‐depleted cells. That is, it seems that in non‐small‐cell lung cancer H460 cells, p53 complexes with PBK, is translocated to cytoplasm, and is subjected to ubiquitination and degradation under exposure of paclitaxel. These findings provide a mechanistic evidence for PBK‐mediated regulation of p53 expression. On the other hand, Mdm2 E3 ubiquitin ligase functions as a negative regulator of p53 and the expression of Mdm2 was induced by p53 [55, 62]. Our findings show that inhibition of Mdm2 elevates both apoptosis and autophagy in response to paclitaxel, which results from an increase in p53 expression. Collectively, we conclude that PBK diminishes paclitaxel‐induced apoptosis or autophagy through negative regulation of p53 activity and expression. Further studies are required for elucidation of mechanistic role of PBK in autophagy of cancer cells in response to various cellular stresses.

## Author contributions

J‐HP and S‐AP conducted experiments, collected data, and analyzed data. YJL performed experiments. HWP provided some reagents and suggestions. SMO designed and supervised the project. All authors reviewed the manuscript.

## Conflict of interest

The authors declare no conflict of interest.
